# Supplementary material for: Seasonal Shifts in Bacterial and Fungal Microbiomes of Leaves and Associated Leaf-Mining Larvae Reveal Persistence of Core Taxa Regardless of Diet
Source: Microbiol Spectr. 2023 Jan 11;11(1):e03160-22. doi: 10.1128/spectrum.03160-22 (PMC9927363; doi:10.1128/spectrum.03160-22)
Supplement: Supplemental file 1 — Supplemental material. Download spectrum.03160-22-s0001.pdf, PDF file, 0.9 MB [file spectrum.03160-22-s0001.pdf]

Supplementary Information for

Seasonal shifts in bacterial and fungal microbiomes of leaves and associated leaf mining larvae reveal persistence of core taxa regardless of diet

Authors: Hana Šigutová, Martin Šigut, Petr Pyszko, Martin Kostovčík, Miroslav Kolařík and Pavel Drozd
Corresponding author: Hana Šigutová
Email: hana.sigutova@osu.cz

This file includes:
Figures S1 to S6
Tables S1, S2, S5, and S6

Other supplementary materials for this manuscript are uploaded separately, and include the following:
Figure S7 (hierarchical taxonomic composition of the leaf and larval bacterial microbiota throughout the season)
Figure S8 (hierarchical taxonomic composition of the leaf and larval fungal microbiota throughout the season)
Table S3 (contaminant bacterial and fungal ASVs)
Table S4 (bacterial and fungal taxa, the number of reads, and the variables entering the analyses)

Fig. S1. Map of **a)** Czechia showing the sampling locality at Central Moravia (Střeň in PLA Litovelské Pomoraví); **b)** sampling plots with individual trees. Map backgrounds were taken from the website FreeWorldMaps.net (https://www.freeworldmaps.net/europe/czechia/czechia-physical-map.jpg) and Mapy.cz (https://mapy.cz/zakladni).

a)

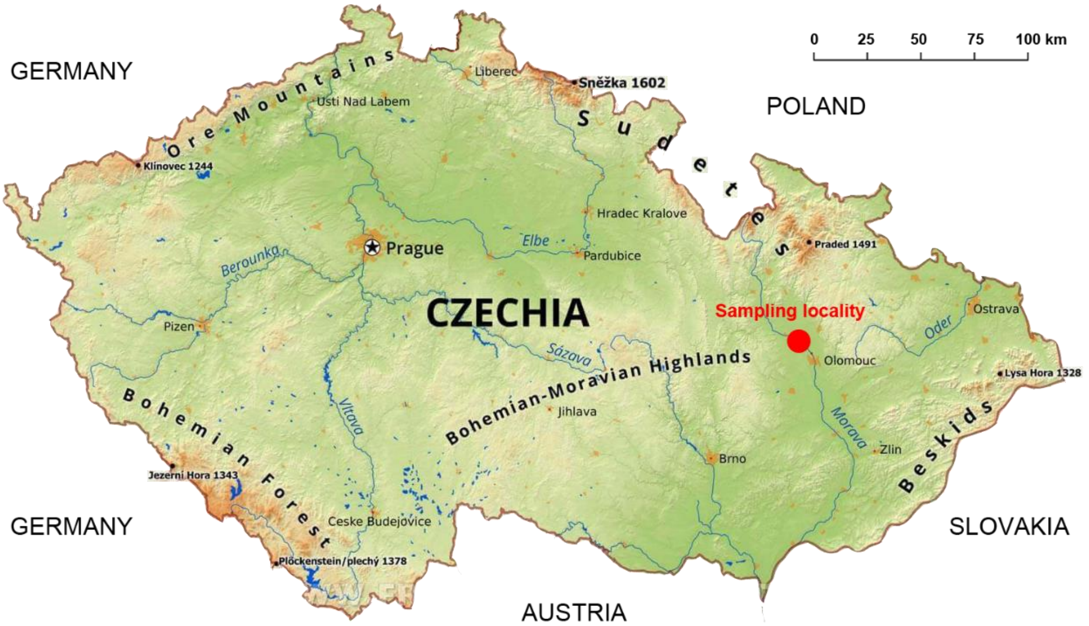

b)

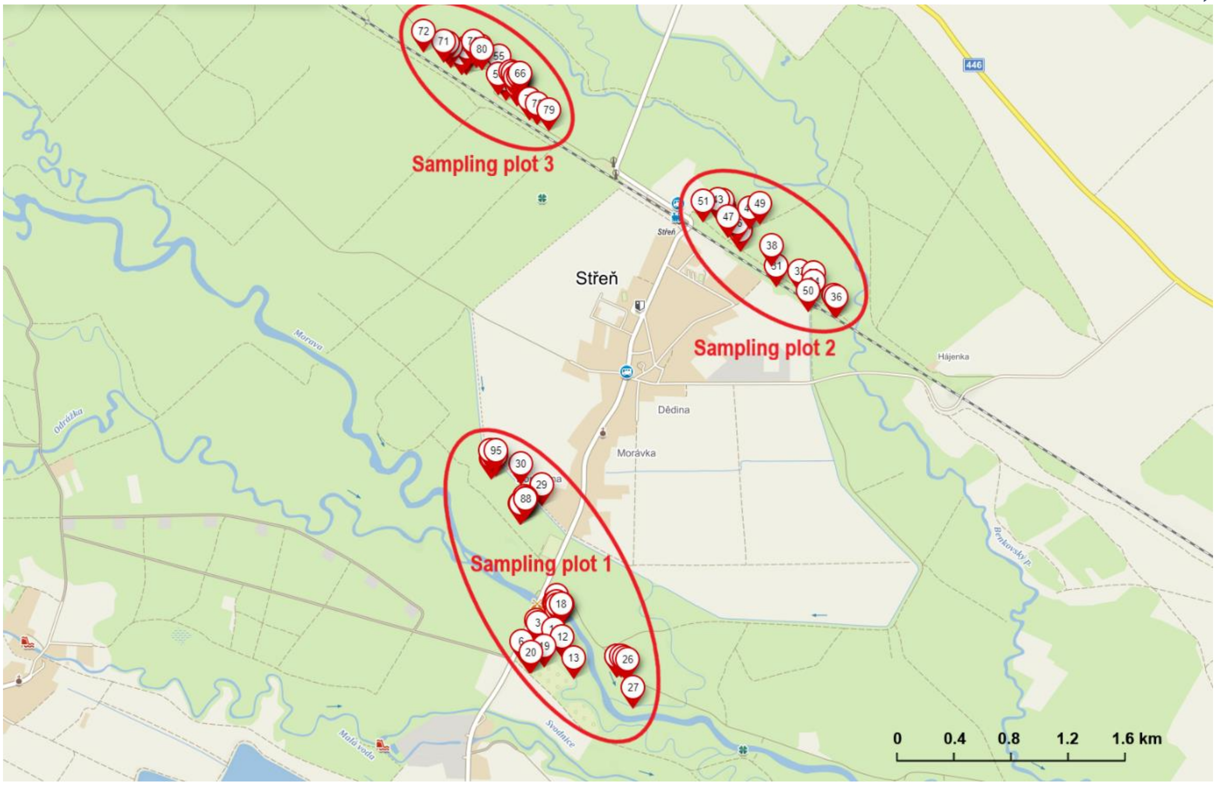

Table S1. Number of leaf-miners and host tree species sampled on individual sampling events.

					Sampling event				
**Type**	**Order**	**Family**	**Species**	**Host tree**	**April**	**June**	**August**	**October**	**Total**
M	Lepidoptera	G	*Phyllonorycter* sp.*			1	6	1	8
M	Lepidoptera	G	*Phyllonorycter coryli* (Nicelli, 1851)	*Corylus avellana*		10	9	8	27
M	Lepidoptera	G	*Phyllonorycter esperella* (Goeze, 1783)	*Carpinus betulus*		10	12	9	31
M	Lepidoptera	G	*Phyllonorycter heegeriella* (Zeller, 1846)	*Quercus robur, Q. petraea*		1		22	23
M	Lepidoptera	G	*Phyllonorycter klemannella* (Fabricius, 1781)	*Alnus glutionsa*			10	5	15
M	Lepidoptera	G	*Phyllonorycter nicellii* (Stainton, 1851)	*Corylus avellana*		9	9	9	27
M	Lepidoptera	G	*Phyllonorycter quercifoliella* (Zeller, 1839)	*Quercus robur, Q. petraea*		27	8	12	47
M	Lepidoptera	G	*Phyllonorycter rajella* (Linnaeus, 1758)	*Alnus glutionsa*		9	6	13	28
M	Lepidoptera	G	*Phyllonorycter roboris* (Zeller, 1839)	*Quercus robur, Q. petraea*		5	13		18
M	Lepidoptera	G	*Phyllonorycter stettinensis* (Nicelli, 1852)	*Alnus glutionsa*		9			9
M	Lepidoptera	G	*Phyllonorycter tenerella* (Joannis, 1915)	*Carpinus betulus*		8	6	9	23
M	Lepidoptera	Ti	*Tischeria ekebladella* (Bjerkander, 1795)	*Quercus robur, Q. petraea*		18	18	19	55
T	Fagales	B	*Alnus glutinosa* (L.) Gaertn.		18	19	19	19	75
T	Fagales	B	*Carpinus betulus* L.		18	18	18	18	72
T	Fagales	B	*Corylus avellana* L.		18	20	18	18	74
T	Fagales	F	*Quercus petraea* (Matt.) Liebl.		18	18	18	18	72
T	Fagales	F	*Quercus robur* L.		19	19	19	17	74
**Total**					91	201	189	197	678

Type: M – leaf-miner, T – tree, Family: G – Gracillariidae, Ti – Tischeriidae, B – Betulaceae, F – Fagaceae

*data for the individuals from *Quercus robur* sampled in low numbers (including *P. harrisella* and *P.muelleriella*) were merged and analyzed at the genus level

Table S2. List of taxonomic resources used for identification of leaf miners.

1. Laštůvka, Z. & Liška, J. (2010) Checklist of Lepidoptera of the Czech Republic (Insecta: Lepidoptera). URL http://lepidoptera.wz.cz
2. Patočka, J. (1980) Die Raupen und Puppen der Eichenschmetterlinge Mitteleuropas. Monographien zur angewandten Entomologie.
3. Ratnasingham, S. & Hebert, P. D. N. (2007) BOLD: The Barcode of Life Data System. *Molecular Ecology Notes* 7, 355–364. URL http://barcodinglife.org
4. De Prins, J. & De Prins, W. (2012) Global Taxonomic Database of Gracillariidae (Lepidoptera). URL http://www.gracillariidae.net
5. Ellis, W. N. (2007) Leafminers and plant galls of Europe, in, Zoölogisch Museum Amsterdam. URL https://bladmineerders.nl
6. Hering, E. M. (1957) Bestimmungstabellen der Blattminen von Europa einschliesslich des Mittelmeerbeckens und der Kanarischen Inseln., Uitgeverij Dr. W. Junk, s'Gravenhage.

Table S5. Explanatory variables (and their interactions) with significant effect on the composition and richness of leaf and larval microbiota. n.s. = the AIC values of their models were higher than of the null model. For leaf microbiomes, we tested the following explanatory variables: tree species, DBH, sampling height, irradiation of crown, sampling plot, and time point. For larval microbiomes, we tested all variables mentioned above plus larval species and family.

	**Bacterial composition**		**Fungal composition**	
	% of variability	statistics	% of variability	statistics
**Leaves**				
Tree species	13.80	df = 361, F = 17.84, P = 0.001***	29.39	df = 362, F = 47.81, P = 0.001***
Time point	13.33	df = 365, F = 68.92, P = 0.001***	5.13	df = 361, F = 33.38, P = 0.001***
Irradiation	0.78	df = 360, F = 4.03, P = 0.001***		n.s.
Sampling plot		n.s.	2.99	df = 359, F = 9.73, P = 0.001***
*Tree species*t. point*	3.26	df = 356, F = 4.22, P = 0.001***	3.90	df = 355, F = 6.34, P = 0.001***
*Tree species*s. plot*		n.s.	5.26	df = 347, F = 4.28, P = 0.001***
**Larvae**				
Tree species		n.s.	3.88	df = 305, F = 3.47, P = 0.001***
Time point	4.66	df = 298, F = 18.79, P = 0.001***	6.82	df = 309, F = 24.39, P = 0.001***
Larval species	17.64	df = 299, F = 6.46, P = 0.001***		n.s.
Larval family		n.s.	0.55	df = 304, F = 1.97, P = 0.008**
*Larval species*t. point*	6.23	df = 288, F = 2.51, P = 0.001***		n.s.
*Tree species*t. point*		n.s.	4.79	df = 300, F = 4.29, P = 0.001***

	**Bacterial richness**	**Fungal richness**
**Leaves**		
Tree species	df = 362, F = 5.69, P < 0.001***	df = 362, F = 22.65, P < 0.001***
Time point	n.s.	df = 360, F = 22.75, P < 0.001***
Sampling plot	n.s.	df = 358, F = 3.62, P = 0.028*
*Tree species*t. point*	n.s.	df = 350, F = 3.23, P = 0.001**
**Larvae**		
Time point	df = 297, F = 17.76, P < 0.001***	df = 308, F = 140.18, P < 0.001***
Larval species	df = 299, F = 29.07, P < 0.001***	df = 297, F = 3.91, P < 0.001***
Tree species	n.s.	df = 295, F = 3.89, P = 0.021*

Fig. S2. PCoA plot based on p-RDA of **a)** bacterial (df = 360, F = 32.30, P = 0.001), and **b)** fungal community composition (df = 360, F = 19.50, P = 0.001) of leaves showing significant shifts as the season progressed. Leaves were sampled at four time points; April, June, August, and October, which corresponds to young, mature, senescent, and old leaves, respectively

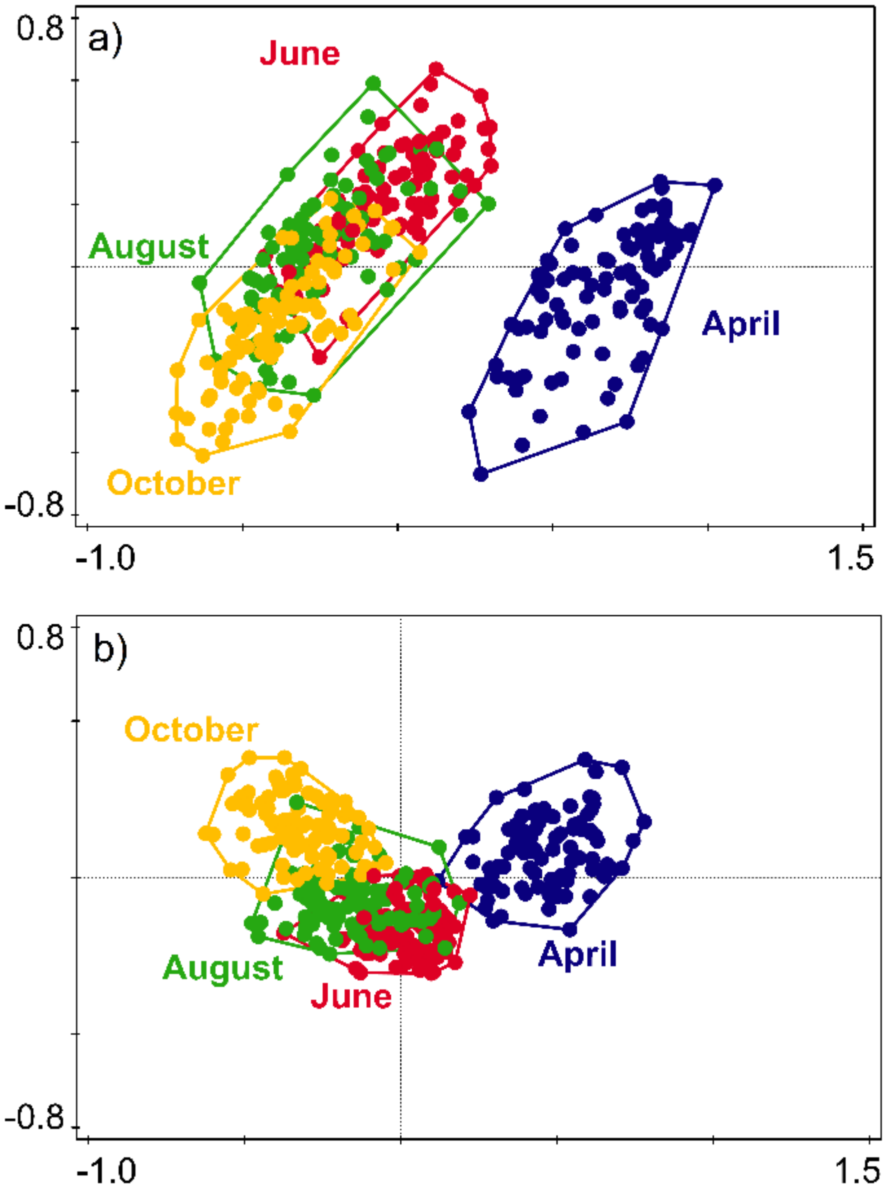

Fig. S3. Rarefied richness of leaf microbiota among tree species for **a)** bacteria (df = 362, F = 5.69, P < 0.001), **b)** fungi (df = 362, F = 22.65, P < 0.001) (mean ± SE) as fitted by GLMs with Gamma distribution.

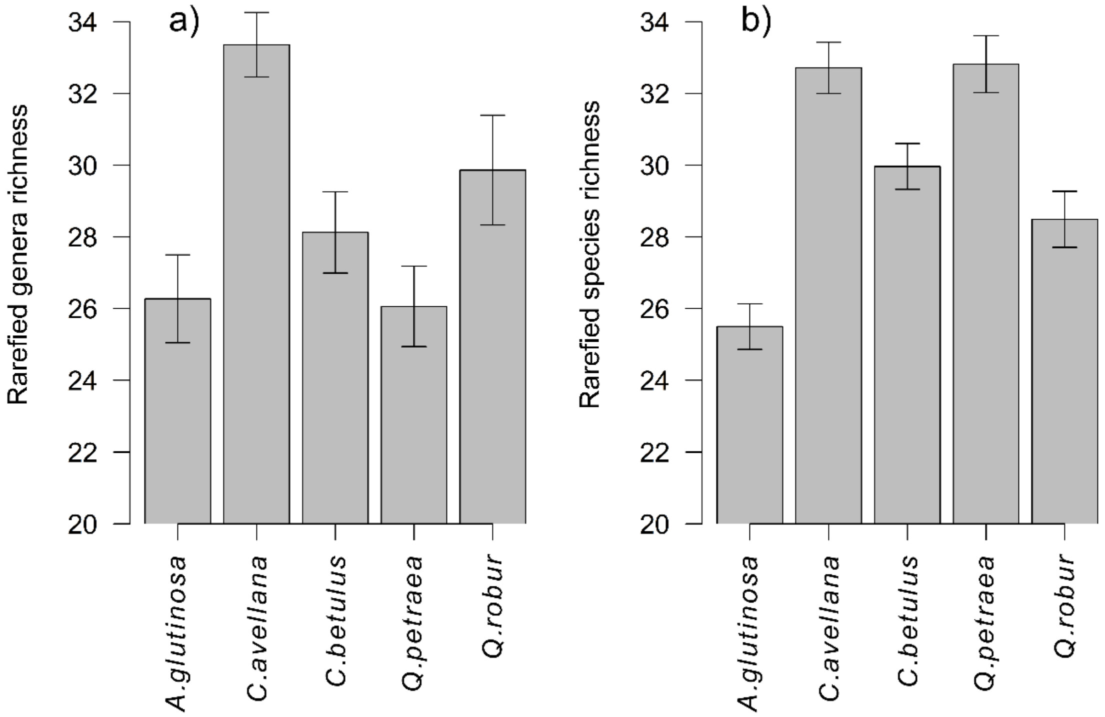

Fig. S4. PCoA showing shifts in composition of larval microbiota due to seasonal progression based on a) p-RDA for bacteria (df = 305, F = 9.90, P = 0.001) and b) p-CCA for fungi (df = 305, F = 20.00, P = 0.001)

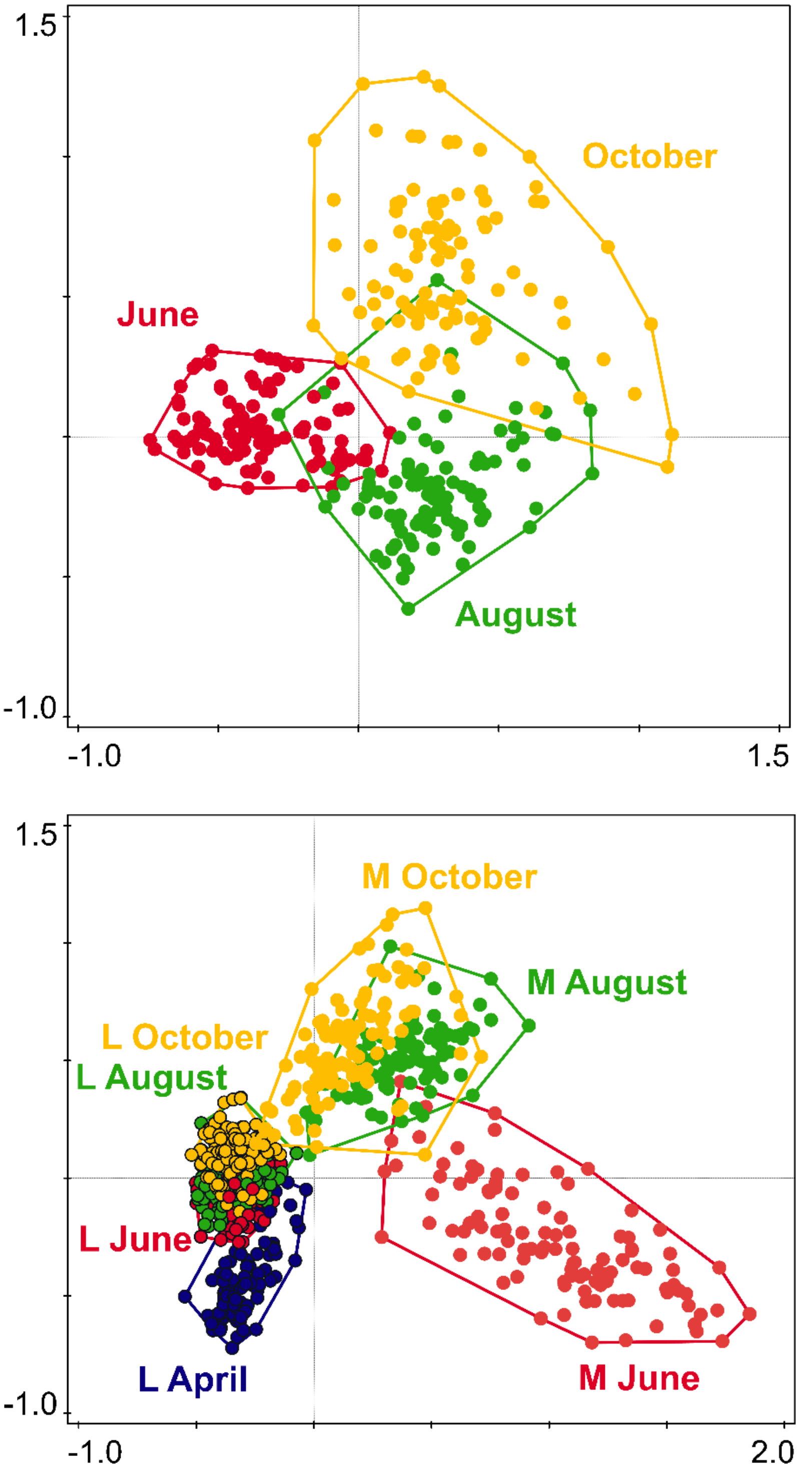

Fig. S5. The p-CCA showing differences in **a)** bacterial (df = 297, F = 2.30, P = 0.001), and **b)** fungal composition of larval microbiota (df = 297, F = 1.60, P = 0.001). Data for *Quercus petraea*, and *Q. robur* were merged for the purposes of this analysis

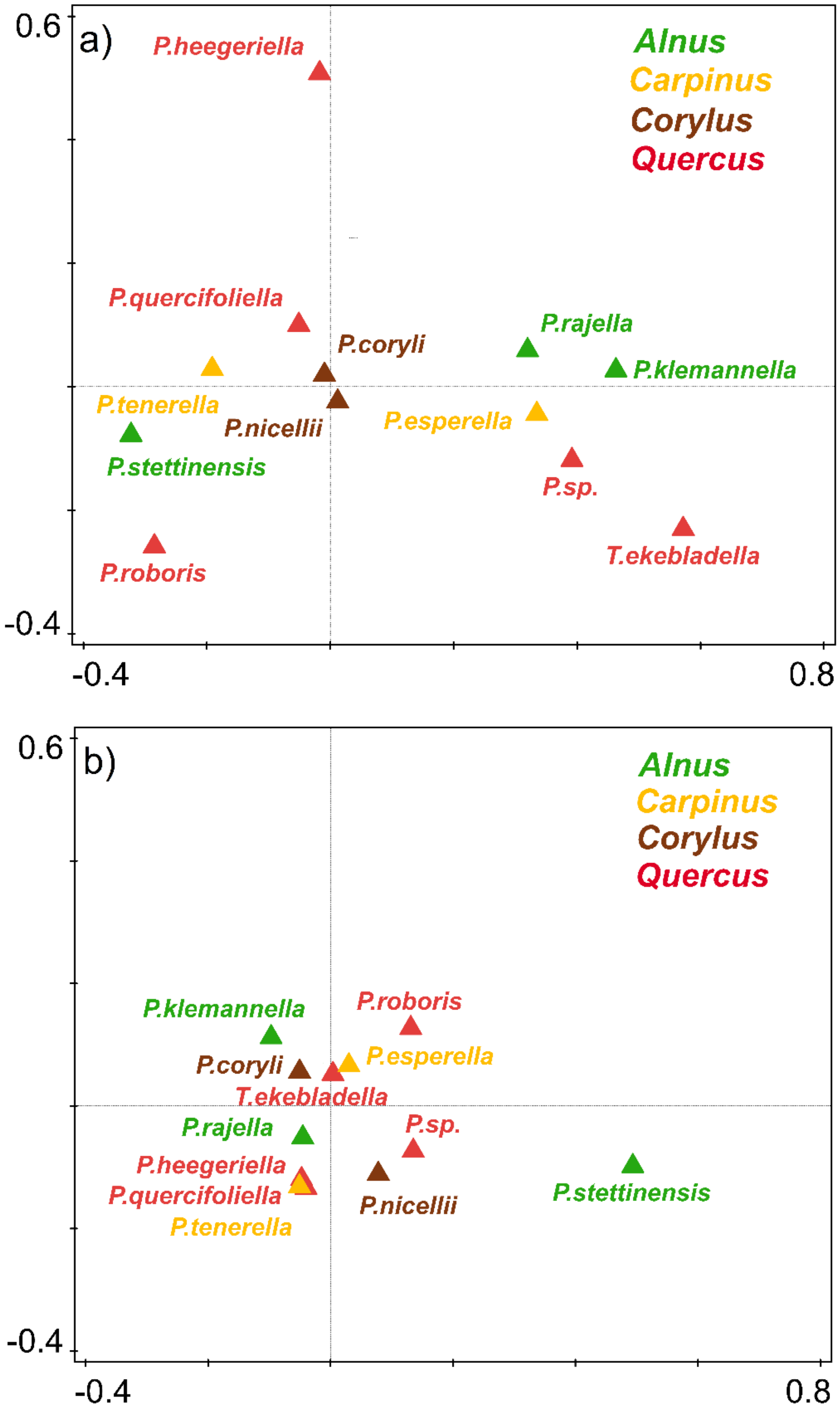

Fig. S6. Rarefied richness among larval species for **a)** bacteria (df = 299, F = 29.07, P < 0.001), **b)** fungi (df = 297, F = 3.91, P < 0.001) as fitted by GLM with Gamma distribution and LM, respectively

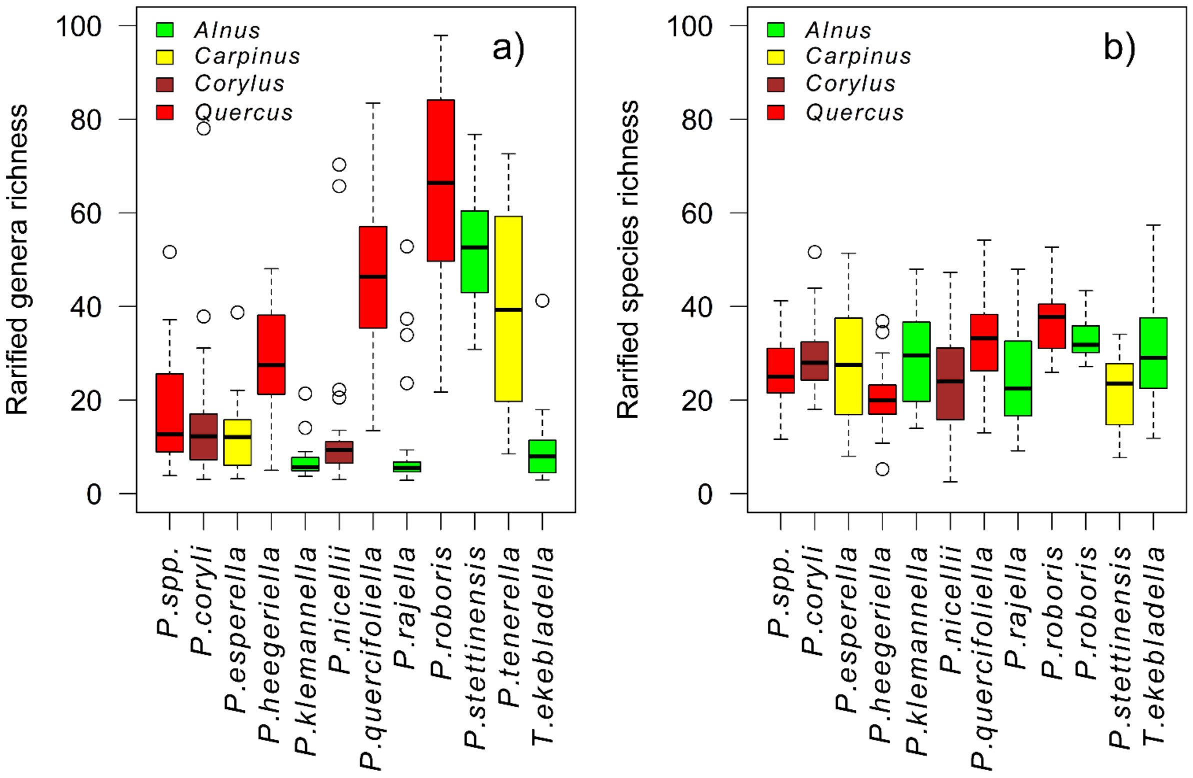

Table S6. Top ten bacterial genera and fungal species with identified seasonal trends. AIC_Δ = difference in AIC of model based simply on differences between leaf and larval samples, and model with added seasonal trend and its interaction with sample type, Sample Type P_{adj} = adjusted p-value from analysis of deviance of the model for differences between leaf and larval samples, Sample Time P_{adj} = adjusted p-value for simple seasonal trend, Interaction P_{adj} = adjusted p-value of interaction between those explanatory variables, Frequency in dataset = Number of samples with presence of given bacteria/fungi, only samples with frequency ≥ 100 were used in the analysis. Taxa occurring in larvae more frequently than predicted by null models are highlighted.

**Bacterial taxon**	**AIC_Δ**	**Sample Type P_{adj}**	**Sample Time P_{adj}**	**Interaction P_{adj}**	**Frequency in dataset**	**Prevalence environment**	**Potential function**	**Reference(s)***
Bacillus	250.54	<0.001	0.200	<0.001	391	leaf/larva	plants: antifungal activity, promotion of plant growth and health; larvae: proteolytic activity counteracting plant protease inhibitors	Bao et al. 2020, Nair et al. 2002, Perissol et al. 1993
Staphylococcus	173.60	<0.001	<0.001	<0.001	354	larva	plants: alleviation of biotic stresses; larvae: protease activity against plant secondary metabolites	Araya et al. 2020, Visôtto et al. 2009
Cutibacterium	155.71	<0.001	<0.001	0.280	417	larva	common core member of arthropod guts, digestive processes in vertebrate guts (carbohydrate fermentation)	Kumar et al. 2020, Koskinioti et al. 2019, Papadopoulos et al. 2020, Perry & Lambert 2006, Wang et al. 2011
Erwinia	134.20	<0.001	<0.001	<0.001	155	leaf	common colonist of leaves	Rastogi et al. 2012
Deinococcus	128.07	<0.001	<0.001	<0.001	246	leaf	promoting plant resistance to environmental stressors (desiccation, pollution, UV radiation, etc.)	Bauermeister et al. 2011, Waight et al. 2007, Díaz-Riaño et al. 2019
P3OB-42	125.85	<0.001	<0.001	<0.001	367	leaf	suppression of phytopathogens	Dobrovol'skaya et al. 2016
Acinetobacter	114.08	<0.001	<0.001	0.228	356	larva	plants: denitrification and organic pollutant degradation; larvae: metabolizing of toxic phenolic glycosides	Bao et al. 2020, Mason et al. 2016
Methylobacterium/Methylorubrum	99.39	<0.001	0.317	<0.001	501	leaf/larva	plants: plant-growth promotion, pathogen resistance; larvae: synthesis of amino acids, production of digestive enzymes and vitamins	Senthilkumar et al. 2015, Ardanov et al. 2012, Jayashree et al. 2011, Martins et al. 2019
Curtobacterium	88.61	<0.001	<0.001	0.044	250	leaf	plant pathogen	Hsieh et al. 2005
Streptococcus	88.50	<0.001	<0.001	0.001	223	larva	degradation of plant secondary metabolites	Osawa et al. 1990, Visôtto et al. 2009
**Fungal taxon**	**AIC_Δ**	**Sample Type P_{adj}**	**Sample Time P_{adj}**	**Interaction P_{adj}**	**Frequency in dataset**	**Prevalence environment**	**Potential function**	**Reference (s)***
Neoascochyta europaea	226.31	<0.001	<0.001	<0.001	154	larva	synthesis of amino acids, oxidative processes	Heisel et al. 2017
Ampelomyces quisqualis	132.18	<0.001	<0.001	0.001	283	larva	suppression of phytopathogens	Falk et al. 1995

Curvibasidium cygneicollum	115.27	<0.001	<0.001	<0.001	185	leaf/larva	plant pathogen	Robert et al. 2019
Botrytis cinerea	106.06	0.999	<0.001	1.000	133	leaf/larva	plant pathogen, regulation of lepidopteran life cycle via sterol synthesis	Elad et al. 2016, Rizvi & Raman 2015
Debaryomyces prosopidis	101.68	<0.001	<0.001	0.685	123	larva	nutrition (carbohydrate assimilation)	Wrent et al. 2014
Ceramothyrium carniolicum	80.49	<0.001	<0.001	<0.001	270	larva	plant pathogen	Nguyen et al. 2019
Mycosphaerelloides madeirae	78.91	<0.001	<0.001	<0.001	102	larva	plant pathogen	Crous et al. 2004
Exobasidium arescens	69.12	<0.001	<0.001	<0.001	104	leaf	plant pathogen	Bergerow et al. 2002
Seimatosporium quercinum	57.32	<0.001	<0.001	<0.001	109	leaf	common colonist of oak twigs	Goonasekara et al. 2016
Candida sake	56.51	<0.001	<0.001	1.000	229	larva	pathogen suppression, nutrition	Malassigné et al. 2021, Usall et al. 2000, Morales et al. 2008

*References are listed on the following pages

References

Araya MA, Valenzuela T, Inostroza NG, Maruyama F, Jorquera MA, Acuña JJ. 2020. Isolation and characterization of cold-tolerant hyper-ACC-degrading bacteria from the rhizosphere, endosphere, and phyllosphere of Antarctic vascular plants. 11. Microorganisms 8:1788.

Ardanov P, Sessitsch A, Häggman H, Kozyrovska N, Pirttilä AM. 2012. *Methylobacterium*-induced endophyte community changes correspond with protection of plants against pathogen attack. PLOS ONE 7:e46802

Bao L, Gu L, Sun B, Cai W, Zhang S, Zhuang G, Bai Z, Zhuang X. 2020. Seasonal variation of epiphytic bacteria in the phyllosphere of *Gingko biloba*, *Pinus bungeana* and *Sabina chinensis*. FEMS Microbiol Ecol 96:fiaa017.

Bauermeister, A., Moeller, R., Reitz, G., Sommer, S., & Rettberg, P. (2011). Effect of relative humidity on *Deinococcus radiodurans*' resistance to prolonged desiccation, heat, ionizing, germicidal, and environmentally relevant UV radiation. Microbial ecology, 61(3), 715-722.

Begerow, D., Bauer, R., & Oberwinkler, F. (2002). The Exobasidiales: an evolutionary hypothesis. Mycological Progress, 1(2), 187-199.

Crous PW, Groenewald JZ, Mansilla JP, Hunter GC, Wingfield MJ (2004). Phylogenetic reassessment of *Mycosphaerella* spp. and their anamorphs occurring on *Eucalyptus*. 1. Stud Mycol 50:195–214

Díaz-Riaño J, Posada L, Acosta IC, Ruíz-Pérez C, García-Castillo C, Reyes A, Zambrano MM. 2019. Computational search for UV radiation resistance strategies in *Deinococcus swuensis* isolated from Paramo ecosystems. PLOS ONE 14:e0221540.

Dobrovol'skaya TG, Khusnetdinova KA, Manucharova NA, Balabko PN. 2016. The structure and functions of bacterial communities in an agrocenosis. Eurasian Soil Sci 49:70–76

Elad Y, Vivier M, Fillinger S. 2016. Botrytis, the Good, the Bad and the Ugly, p. 1–15. In Fillinger, S, Elad, Y (eds.), *Botrytis* – the Fungus, the Pathogen and its Management in Agricultural Systems. Springer International Publishing, Cham

Falk SP, Gadoury DM, Cortesi P, Pearson RC, Seem RC. 1995. Parasitism of *Uncinula necator* cleistothecia by the mycoparasite *Ampelomyces quisqualis*. Phytopathology 85:794–800.

Goonasekara I, Maharachchikumbura S, Wijayawardene N, Phookamsak R, Schumacher R, Bahkali A, Jones E, Hyde K. (2016). *Seimatosporium quercina* sp. nov. (Discosiaceae) on *Quercus robur* from Germany. Phytotaxa 255:240–248

Heisel T, Montassier E, Johnson A, Al-Ghalith G, Lin Y-W, Wei L-N, Knights D, Gale CA. 2017. High-fat diet changes fungal microbiomes and interkingdom relationships in the murine gut. mSphere 2:e00351-17

Hsieh TF, Huang HC, Mündel H-H, Conner RL, Erickson RS, Balasubramanian PM. 2005. Resistance of common bean (*Phaseolus vulgaris*) to bacterial Wilt Caused by *Curtobacterium flaccumfaciens* pv. *flaccumfaciens.* J Phytopathol 153:245–249

Jayashree S, Lalitha R, Vadivukkarasi P, Kato Y, Seshadri S. 2011. Cellulase production by pink pigmented facultative methylotrophic strains (PPFMs). Appl Biochem Biotechnol 164:666–680

Koskinioti, P., Ras, E., Augustinos, A. A., Tsiamis, G., Beukeboom, L. W., Caceres, C., & Bourtzis, K. (2019). The effects of geographic origin and antibiotic treatment on the gut symbiotic communities of *Bactrocera oleae* populations. Entomologia Experimentalis et Applicata, 167(3), 197-208.

Kumar, V., Tyagi, I., Tyagi, K., & Chandra, K. (2020). Diversity and structure of bacterial communities in the gut of spider: Thomisidae and Oxyopidae. Frontiers in Ecology and Evolution, 8, 588102.

Malassigné, S., Minard, G., Vallon, L., Martin, E., Valiente Moro, C., & Luis, P. (2021). Diversity and functions of yeast communities associated with insects. Microorganisms, 9(8), 1552.

Martins AM, Latham JA, Martel PJ, Barr I, Iavarone AT, Klinman JP. 2019. A two-component protease in *Methylorubrum extorquens* with high activity toward the peptide precursor of the redox cofactor pyrroloquinoline quinone. J Biol Chem 294:15025–15036

Mason CJ, Lowe-Power TM, Rubert-Nason KF, Lindroth RL, Raffa KF. 2016. Interactions between bacteria and aspen defense chemicals at the phyllosphere–herbivore interface. J Chem Ecol 42:193–201

Morales, H., Sanchis, V., Usall, J., Ramos, A. J., & Marín, S. (2008). Effect of biocontrol agents *Candida sake* and *Pantoea agglomerans* on *Penicillium expansum* growth and patulin accumulation in apples. International journal of food microbiology, 122(1-2), 61-67

Nair J r., Singh G, Sekar V. 2002. Isolation and characterization of a novel *Bacillus* strain from coffee phyllosphere showing antifungal activity. J Appl Microbiol 93:772–780.

Nguyen D, Boberg J, Ihrmark K, Stenström E, Stenlid J. 2016. Do foliar fungal communities of Norway spruce shift along a tree species diversity gradient in mature European forests? Fungal Ecol 23:97–108

Osawa R, Mitsuoka T. 1990. Selective medium for enumeration of tannin-protein complex-degrading *Streptococcus* spp. in feces of koalas. Appl Environ Microbiol 56:3609–3611.

Papadopoulos, C., Karas, P. A., Vasileiadis, S., Ligda, P., Saratsis, A., Sotiraki, S., & Karpouzas, D. G. (2020). Host species determines the composition of the prokaryotic microbiota in *Phlebotomus* sandflies. Pathogens, 9(6), 428.

Perissol C, Roux M, Le Petit J. 1993. Succession of bacteria attached to evergreen oak leaf surfaces [phylloplane]. Eur J Soil Biol 29:167–176.

Perry, A. L., and Lambert, P. A. (2006). *Propionibacterium acnes*. Lett. Appl. Microbiol. 42, 185–188. doi: 10.1111/j.1472-765X.2006.01866.x

Rastogi G, Sbodio A, Tech JJ, Suslow TV, Coaker GL, Leveau JHJ. 2012. Leaf microbiota in an agroecosystem: spatiotemporal variation in bacterial community composition on field-grown lettuce. 10. ISME J 6:1812–1822.

Rizvi SZM, Raman A. 2015. Epiphyas postvittana (Lepidoptera: Tortricidae)—*Botrytis cinerea* (Helotiales: Sclerotiniaceae)—*Vitis vinifera* (Vitales: Vitaceae) interaction: the role of *B. cinerea* on the development of E. postvittana in synthetic nutritional media. J Econ Entomol 108:1646–1654

Robert K, Baranowska M, Behnke-Borowczyk J. 2019. The effect of size of black cherry stumps on the composition of fungal communities colonising stumps. Open Life Sci 14:482–493

Senthilkumar M, Madhaiyan M, Sundaram SP, Kannaiyan S. 2009. Intercellular colonization and growth promoting effects of *Methylobacterium* sp. with plant-growth regulators on rice (*Oryza sativa* L. Cv CO-43). Microbiol Res 164:92–104

Usall, J., Teixidó, N., Fons, E., & Vinas, I. (2000). Biological control of blue mould on apple by a strain of *Candida sake* under several controlled atmosphere conditions. International Journal of Food Microbiology, 58(1-2), 83-92

Visôtto LE, Oliveira MGA, Ribon AOB, Mares-Guia TR, Guedes RNC. 2009. Characterization and identification of proteolytic bacteria from the gut of the velvetbean caterpillar (Lepidoptera: Noctuidae). Environ Entomol 38:1078–1085.

Waight K, Pinyakong O, Luepromchai E. 2007. Degradation of phenanthrene on plant leaves by phyllosphere bacteria. J Gen Appl Microbiol 53:265–272.

Wang, Y., Gilbreath, T. M., Kukutla, P., Yan, G., and Xu, J. (2011). Dynamic gut microbiome across life history of the malaria mosquito *Anopheles gambiae* in Kenya. PLoS One 6:e24767. doi: 10.1371/journal.pone.024767

Wrent, P., Rivas, E.M., Gil de Prado, E., Peinado, J.M., de Silóniz, M.I., 2014. *Debaryomyces,* in: Batt, C.A., Tortorello, M.L. (Eds.), Encyclopedia of Food Microbiology (Second Edition). Academic Press, Oxford, pp. 563–570. https://doi.org/10.1016/B978-0-12-384730-0.00081-1
